# Sex-specific adaptive immune responses in spinal cord injury: Observations across species

**DOI:** 10.4103/NRR.NRR-D-25-00532

**Published:** 2025-08-13

**Authors:** Reena Kumari, John C. Gensel

**Affiliations:** Spinal Cord and Brain Injury Research Center, Department of Physiology, University of Kentucky College of Medicine, Lexington, KY, USA

Biological sex is increasingly recognized as a crucial factor in evaluating the translational value of preclinical spinal cord injury (SCI) studies. The rising incidence of SCI in females challenges the historical precedent of SCI being a male-dominated condition. In contrast, most basic science researchers utilize single-sex studies to minimize complications associated with bladder care in males (Stewart et al., 2020). The findings of our recent publication identify sexually dimorphic immune responses to SCI in both mice and pigs (Kumari et al., 2025). Here, we will highlight these findings and discuss the impact of sex on SCI inflammation and recovery.

The inflammatory response after SCI is intricate and involves innate and adaptive immune systems, several cell types, and inflammatory cytokines. Although the immune response can have some positive effects, the widespread inflammatory process is believed to play a major role in neural degeneration. Our previous findings indicate that the inflammatory response after SCI is sex-dependent, affecting cellular recruitment and phenotype (Stewart et al., 2021). Specifically, we observed sex-dependent inflammatory profiles acutely after SCI, with females having more infiltrating monocyte-derived macrophages than microglia and neutrophils, as well as males having increased microglial activation in response to SCI. Secondary injury mechanisms also vary, with females exhibiting higher levels of CYBB (NOX2), a marker associated with reactive oxygen species, as well as MRC1 (CD206), a gene linked to reparative phenotypes. In contrast, males show a greater prevalence of complement-driven pathology, characterized by C1qa complement expression (Stewart et al., 2021).

In our more recent work (Kumari et al., 2025), we sought to explore histological changes, including inflammation, after SCI in a pig model. Large animal models, such as pigs, are gaining popularity in SCI research due to their anatomical and physiological similarities to humans. However, comparative research between rodent and pig SCI models is limited. Examining pig spinal cord tissue 6 weeks after injury, we observed ramified astrocytic processes within areas of frank tissue pathology, a phenomenon less common in rodents but seen in humans and non-human primates (Kumari et al., 2025). This observation suggests pigs may be a valuable model for therapies targeting the glial scar. We also reported that T-cell responses differ between male and female pigs 6 weeks post-SCI. Specifically, we observed that the density of intraspinal CD3^+^ T-cells was significantly increased in females relative to males. Interestingly, we confirmed the same increase in CD3^+^ intraspinal T-cell accumulation in female mice, relative to males, at the same time point after SCI (Kumari et al., 2025). We did not observe differences in astrocytes, microglia/macrophages, neutrophils, and B cells between male and female pigs at 6 weeks post-injury. Given the role of inflammation in secondary injury, pain, and recovery, these findings have significant implications for translational neurotrauma research.

T-lymphocytes, though fewer than macrophages or activated microglia after SCI, can modulate secondary injury and pathophysiology. They recognize specific antigens such as myelin basic protein, proliferate, and produce pro-inflammatory cytokines and growth factors. Central nervous system–reactive T-cells worsen axonal injury, demyelination, and functional deficits following experimental SCI. As seen after traumatic brain injury and SCI, the adaptive immune system can exacerbate tissue damage but also promote central nervous system repair. For these reasons, the role of autoimmunity in SCI is still debated (Ankeny and Popovich, 2009).

Current research focuses on harnessing autoimmune-mediated repair while minimizing the pathological effects of autoreactive lymphocytes without compromising host defense. The key challenge is identifying the molecular factors that regulate these opposing functions to develop effective treatments (Ankeny and Popovich, 2009). Our most recent findings implicate sex as a key influence in autoimmunity after SCI.

T-cells can have either pro- or anti-nociceptive effects. T-cell-derived factors act on pre- and post-synaptic nerve terminals to enhance neurotransmission and mediate central pain sensitization. Individuals with chronic neuropathic pain exhibit distinct T-cell phenotypic profiles compared to control populations (Trivedi et al., 2006). In mice, CD4^+^ and CD8^+^ T-cells show a biphasic infiltration pattern, with peaks occurring between 7–14 days and again at 42 days (Trivedi et al., 2006). However, CD8^+^ T-cells infiltrate in higher numbers and tend to cluster in the fibrosis zone (Trivedi et al., 2006). It is believed that CGRP from nociceptive neurons stimulates interleukin-23 production in dermal dendritic cells, which then activates γδ T-cells and promotes interleukin-17 production (Baral et al., 2019). Additionally, CGRP can interact with RAMP1 on dendritic cells to either reduce Th1 cell differentiation or enhance dendritic cell polarization towards Th2 cell differentiation. These elements of the adaptive immune response are crucial in SCI complications including pain.

T-cells have been implicated in pain development in the spared nerve injury model of peripheral neuropathic pain (Costigan et al., 2009). Interestingly, we previously observed that pioglitazone, an antidiabetic with analgesic properties, alleviated SCI pain–like responses in female but not male mice (McFarlane et al., 2020). One mechanism of action for pioglitazone is T-cell modulation. Our findings of increased T-cells in female mice and pigs implicate T-cells as potential contributors to sex-specific pain responses after SCI (Kumari et al., 2025). Most SCI pain studies have focused on young male rodents, reflecting the higher male prevalence in SCI patients. However, unlike other pain conditions, neuropathic pain prevalence and intensity in SCI are similar between sexes, with females potentially experiencing higher overall pain, including nociceptive pain. Growing experimental evidence suggests that males and females may process pain differently, highlighting the need for sex-specific treatment strategies.

Sexual dimorphism is crucial in neuropathic pain, with distinct outcomes observed in both humans and rodents. The development of neuropathic pain is strongly linked to spinal cord inflammation in a sex-specific manner (Fiore et al., 2023). This, coupled with observations of sexual dimorphisms in intraspinal inflammation after SCI, sheds light on sex differences in neuropathology and complications like pain. A dimorphic immune response likely contributes to these comorbidities and should be considered in translational research and clinical applications. Furthermore, studies suggest that the humoral response and lymphocytes play a role in tissue damage and exacerbate central nervous system degeneration after SCI (Klein and Flanagan, 2016). Despite growing attention, the mechanisms underlying these sexual dimorphisms are not well understood and may involve both adaptive and innate immune responses and factors beyond immune responses.

**Figure 1:**
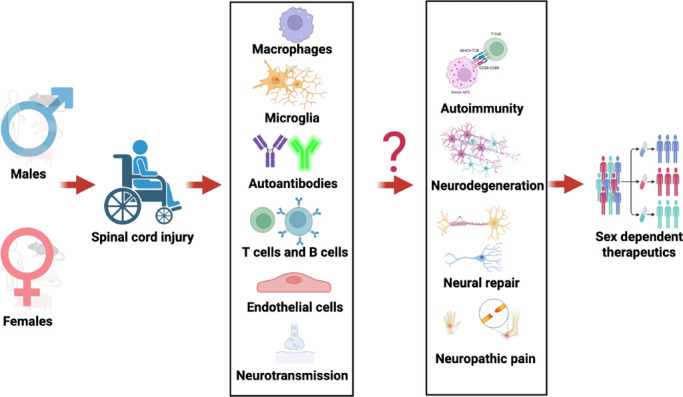
Mechanisms and contributors of sexual dimorphisms after spinal cord injury. Sexual dimorphisms present before spinal cord injury lead to differences in immune responses after the injury and influence various aspects of secondary injury progression. Created with BioRender.com.

Sex differences in innate and adaptive immune responses are evolutionarily conserved across species, varying with age and reproductive status (Klein and Flanagan, 2016). Our data suggest that sex is a critical factor in immune-targeted SCI therapies, emphasizing the need for sex-stratified clinical and preclinical studies. Therefore, considering sex is essential for interpreting and generalizing research findings which require identifying the molecular factors that regulate these opposing actions to develop effective treatments (**Figure 1**). This dimorphism is driven by factors not yet fully understood. Gaining a deeper understanding of sexual dimorphism in both adaptive and innate immune responses is crucial for addressing autoimmunity, neurodegeneration, repair processes, neuropathic pain, and analgesic efficacy, ultimately improving the effectiveness of neuroprotective treatments for both sexes.

*This work was supported by University of Kentucky-SCoBIRC Endowed Chair #5; Craig H. Neilsen 1173493 and NIH RF1NS135504 (to JCG).*
